# Transcriptome-Wide Identification and Expression Profiling of the DOF Transcription Factor Gene Family in *Chrysanthemum morifolium*

**DOI:** 10.3389/fpls.2016.00199

**Published:** 2016-02-23

**Authors:** Aiping Song, Tianwei Gao, Peiling Li, Sumei Chen, Zhiyong Guan, Dan Wu, Jingjing Xin, Qingqing Fan, Kunkun Zhao, Fadi Chen

**Affiliations:** College of Horticulture, Nanjing Agricultural UniversityNanjing, China

**Keywords:** *Chrysanthemum morifolium*, DNA binding with one finger, phylogenetic analysis, stress response, transcription factors

## Abstract

The family of DNA binding with one finger (DOF) transcription factors is plant specific, and these proteins contain a highly conserved domain (DOF domain) of 50-52 amino acids that includes a C2C2-type zinc finger motif at the N-terminus that is known to function in a number of plant processes. Here, we characterized 20 *DOF* genes in the important ornamental species chrysanthemum (*Chrysanthemum morifolium*) based on transcriptomic sequences. Phylogenetic analysis identified one pair of putative orthologous proteins in *Arabidopsis* and chrysanthemum and six pairs of paralogous proteins in chrysanthemum. Conserved motifs in the DOF proteins shared by *Arabidopsis* and chrysanthemum were analyzed using MEME. Bioinformatics analysis revealed that 13 *CmDOFs* could be targeted by 16 miRNA families. Moreover, we used 5' RLM-RACE to map the cleavage sites in *CmDOF3, 15*, and *21*. The expression of these 20 genes in response to phytohormone treatments and abiotic stresses was characterized, and the expression patterns of six pairs of paralogous *CmDOF* genes were found to completely differ from one another, except for *CmDOF6* and *CmDOF7*. This work will promote our research of the various functions of *DOF* gene family members in plant hormone and stress responses.

## Introduction

Transcription factors (TFs) that determine gene transcription rates can be bound to *cis*-regulatory elements of promoters. Many TFs can be divided into different gene families according to their conserved domains. The DNA binding with one finger (DOF) TF family is plant specific and contains a conserved DOF domain (Yanagisawa, 2002). The DOF domain structure includes a C2C2 zinc finger that contains 50-52 amino acid residues; this zinc finger specifically binds to an element with the sequence 5′-AAAG-3′ (Yanagisawa, 2004). In addition to the DNA-binding domain, DOF TF proteins contain a bipartite nuclear localization signal (NLS) that partly overlaps with the conserved DOF DNA-binding domain (Krebs et al., 2010) and a C-terminal transcriptional activation domain (Yanagisawa, 2001). Moreno-Risueno et al. (2007) grouped the DOF family into seven subfamilies based on tree topology and corresponding phylogenetic relationships that probably originated from gene duplication events from a paraphyletic basal grade.

Since the first DOF protein, ZmDOF1, was identified in maize (Yanagisawa and Izui, 1993), additional DOF proteins have been found in many other plants (Cai et al., 2013; Negi et al., 2013). DOF TFs play multiple roles in different biological processes, such as flowering time (Fornara et al., 2009; Wei et al., 2010), seed protein content and color (Gupta et al., 2011), carbon and nitrogen metabolism (Yanagisawa et al., 2004; Tanaka et al., 2009), germination (Isabel-Lamoneda et al., 2003), light-mediated regulation (Park et al., 2003), vascular system development (Le Hir and Bellini, 2013), seed storage protein accumulation (Gaur et al., 2011), and hormone response (Gabriele et al., 2010), in various plants. For example, *AtDOF4.1* overexpression lines showed severe growth retardation and delayed stem bolting and flowering, suggesting that *AtDOF4.1* might act as a transcriptional repressor in the regulation of flowering time (Ahmad et al., 2013). *AtDOF4.2* might regulate shoot branching through the up-regulation of three branching-related genes and seed epidermis development through the direct binding and activation of the cell wall loosening-related gene *AtEXPA9* in *Arabidopsis* (Zou et al., 2013). *In silico cis*-regulatory element analysis indicated that *SbDOF* genes might be involved in light responsiveness, endosperm-specific gene expression, hormone responsiveness, meristem-specific expression and the stress response (Kushwaha et al., 2011). DOF TFs in *Arabidopsis* also play a unique role in vascular development and function (Le Hir and Bellini, 2013).

Recently, an increasing number of studies have shown that DOF TFs are also involved in the regulation of biotic and abiotic stress responses (Corrales et al., 2014; Ma et al., 2015; Sasaki et al., 2015). Thirty-five DOF full-length cDNAs were recently identified in the potato genome, and many *StDOF* genes were detected in various organs; several of these genes were up-regulated by abscisic acid (ABA) and abiotic stresses, such as drought and salinity (Venkatesh and Park, 2015). Similarly, several *ZmDOF* genes were up-regulated during salt treatment of seedlings (Chen and Cao, 2014). Overexpression of *BBF1* from tobacco stimulates the transcription of the tobacco mosaic virus resistance gene *N* and defense-related responses, including ROS production (Takano et al., 2013). The identification of *DOF* genes associated with newer functions, such as abiotic stresses, needs to be explored for crop improvement (Gupta et al., 2015).

Chrysanthemum (*Chrysanthemum morifolium*), one of the four most famous cut flowers in the world, is susceptible to various biotic and abiotic stresses (An et al., 2014). To our knowledge, little information has been reported on the isolation and functional analysis of DOF TFs in chrysanthemum. Here, we isolated 20 DOF TFs in chrysanthemum based on a set of transcriptomic data. We performed a comparative phylogenetic analysis of chrysanthemum and *Arabidopsis* genes *in silico* and investigated the transcript levels in response to various phytohormones and abiotic stresses using qRT-PCR. Moreover, *CmDOF3, 15*, and *21* were confirmed as real targets of miRNA in plants by 5′ RNA ligase-mediated rapid amplification of cDNA ends (5′ RLM-RACE). The results provide novel insights into the stress responses of *CmDOF* genes and promote a better understanding of the structure and function of DOFs in chrysanthemum.

## Materials and methods

### Plant materials and growth conditions

Cuttings of the cut flower chrysanthemum cultivar “Jinba,” maintained by the Chrysanthemum Germplasm Resource Preservation Centre (Nanjing Agricultural University, Nanjing, China), were rooted in vermiculite in the absence of fertilizer in a greenhouse. After 14 days, the cuttings were transplanted to their corresponding growth substrates and then subjected to a range of stress and phytohormone treatments.

### Database searches and sequencing of full-length *CmDOF* cDNAs

All of the putative DOF proteins were retrieved from *C. morifolium* transcriptome data (Zhang et al., 2014). *Arabidopsis* DOF protein sequences were downloaded from The Arabidopsis Information Resource (TAIR) database. The DOF domain sequences of *Arabidopsis* were used as query sequences to identify CmDOF proteins. Multiple alignments among the identified *CmDOF* sequences were also performed to avoid repetition. Furthermore, the full open reading frames of *CmDOF*s were obtained via RACE PCR. The first cDNA strand was synthesized using the dT adaptor primer dT-AP and then subjected to nested PCR using the primer pair CmDOFx-3-F1/F2 and the adaptor primer AP (Table S1). Finally, twenty pairs of gene-specific primers (Table S2) were designed to amplify the full open reading frame sequences. The amplicons were purified using an AxyPrep DNA Gel Extraction Kit (Axygen, Hangzhou, China) and cloned into pMD19-T (TaKaRa, Tokyo, Japan) for sequencing.

### Phylogenetic tree construction and sequence analysis

A phylogenetic tree was constructed with MEGA version 6.0 using the neighbor-joining method (Tamura et al., 2013). Multi-sequence alignments of DOF TFs were performed between *Arabidopsis* and *C. morifolium* using ClustalW software (Larkin et al., 2007). Computation of the theoretical isoelectric point (pI) and molecular weight (Mw) of CmDOF proteins was performed using the Compute pI/Mw online tool (http://web.expasy.org/compute_pi/), and ProtComp 9.0 and PSORT were used to predict their subcellular localization. The MEME v4.10.2 program (Bailey et al., 2015) was employed to identify the motifs present in the CmDOF proteins using the parameter settings suggested by Song et al. (2014b). Target prediction for miRNA was performed using the psRNATarget online tool (Dai and Zhao, 2011).

### Target validation by RLM-RACE

To confirm the predicted targets, RLM-RACE was performed using a FirstChoice RLM-RACE Kit (Ambion, Austin, TX, USA) following the methods described by Song et al. (2015). The RLM-RACE primer and gene-specific primers are shown in Table S5. The RLM-RACE products were purified using an Agarose Gel DNA Purification Kit (TaKaRa), ligated into the pMD19-T vector (TaKaRa), and sequenced.

### Plant treatments

The tissue-specific and treatment-induced transcription profiles of 20 *CmDOF* genes were explored in young seedling roots, stems and leaves as well as in the tube and ray florets of inflorescences at the bud stage and pollen. A variety of abiotic stresses was imposed, including high salinity (200 mM NaCl) and moisture deficit (20% w/v polyethylene glycol 6000, PEG 6000) (Song et al., 2012).

For the NaCl and PEG 6000 assays, young plants were transferred to liquid medium containing the stress agent, and the second true leaves were sampled at various time points (Song et al., 2014a). Other seedlings were subjected to a period of exposure at either 4°C or 40°C in a chamber providing a 16 h photoperiod and 50 μmol· m^−2^·s^−1^ of light, after which the second true leaves were sampled (Song et al., 2014c). The wounding treatment involved cutting the second true leaf. The phytohormone treatments involved spraying the leaves with either 50 μM ABA, 1 mM methyl jasmonate (MeJA) or 200 μM salicylic acid (SA) (Song et al., 2014b). Plants were sampled prior to stress treatment and then after 1, 4, 12, and 24 h.

After sampling, all of the collected material was snap frozen in liquid nitrogen and stored at −70°C. Each treatment was replicated three times.

### Real-time quantitative PCR (qPCR)

Total RNA was isolated from samples using the RNAiso reagent (TaKaRa), according to the manufacturer's instructions; the RNA was then treated with RNase-free DNase I (TaKaRa) to remove potential genomic DNA contamination. The first cDNA strand was synthesized from 1 μg of total RNA using SuperScript III reverse transcriptase (Invitrogen, Carlsbad, CA, USA) according to the manufacturer's instructions. The qPCR was performed using a Mastercycler ep realplex instrument (Eppendorf, Hamburg, Germany). Each 20 μL amplification reaction contained 10 μL of SYBR® Premix Ex Taq™ II (TaKaRa), 0.4 μL of each primer (10 μM), 4.2 μL of H_2_O and 5 μL of cDNA template. The PCR cycling regime consisted of an initial denaturation (95°C/2 min) followed by 40 cycles of 95°C/10, 55°C/15, and 72°C/20 s. A melting curve analysis was conducted following each assay to confirm the specificity of the amplicons. Gene-specific primers (sequences shown in Table S3) were designed using Primer3 Release 2.3.4 (Rozen and Skaletsky, 2000), and the *EF1*α gene was employed as a reference sequence. Relative transcript abundances were calculated via the 2^−ΔΔCT^ method (Livak and Schmittgen, 2001). Three independent experiments were conducted.

### Data analysis

The relative transcript expression levels of each *CmDOF* were log_2_ transformed. The profiles were compared using Cluster v3.0 software (De Hoon et al., 2004) and visualized using Treeview (Eisen et al., 1998). SPSS v17.0 software (SPSS Inc., Chicago, IL, USA) was employed for all statistical analyses.

## Results

### Phylogenetic relationships among DOF proteins of chrysanthemum

The 20 isolated *DOF* sequences were designated as *CmDOF1* through *CmDOF21*, except CmDOF17, whose DOF domain is lost. The full-length cDNAs varied in length from 645 to 1817 bp, and their predicted protein products were composed of between 166 and 453 amino acid residues. Details regarding the *CmDOF* sequences are given in Table 1. Fifteen CmDOF proteins were predicted to show nuclear localization, excluding CmDOF7, 9, 10, 13, and 15. The conserved bipartite NLS was not found in the latter six proteins, which were predicted as being localized to the cytoplasm based on PSORT analysis.

**Table 1:** **Summary of *CmDOF* sequences and the identities of likely *A. thaliana* homologs**.

**Gene**	**GenBank Accession No**.	**Amino Acids Length (aa)**	**AtDOF Homologs**	**Locus Name**	**PI**	**MW**	**Subcellular Localization**
*CmDOF1*	KT235675	453	*AtDOF5.2 CDF2*	AT5G39660	5.25	49666.82	N(8.87)
*CmDOF2*	KT235676	286	*AtDOF5.1 CDF1*	AT5G62430	8.78	31987.35	N(8.91)
*CmDOF3*	KT235677	407	*AtDOF3.3 CDF3*	AT3G47500	6.76	45041.56	N(7.59)
*CmDOF4*	KT235678	166	*AtDOF2.3*	AT2G34140	9.07	18884.41	N(8.34)
*CmDOF5*	KT235679	231	*AtDOF3.7 DAG1*	AT3G61850	9.33	26034.92	N(6.40)
*CmDOF6*	KT235680	279	*AtDOF4.6*	AT4G24060	9.44	30217.6	N(7.82)
*CmDOF7*	KT235681	244	*AtDOF5.3 TMO6*	AT5G60200	8.49	27393.72	E(4.60)
*CmDOF8*	KT235682	308	*AtDOF4.6*	AT4G24060	8.19	33548.21	N(7.76)
*CmDOF9*	KT235683	325	*AtDOF1.4*	AT1G28310	9	35686.24	E(5.31)
*CmDOF10*	KT235684	294	*AtDOF1.4*	AT1G28310	8.84	32249.43	E(3.02)
*CmDOF11*	KT235685	311	*AtDOF4.6*	AT4G24060	8.64	33625.05	N(7.33)
*CmDOF12*	KT235686	260	*AtDOF3.6 OBP3*	AT3G55370	9.55	28087.08	N(6.26)
*CmDOF13*	KT235687	339	*AtDOF3.6 OBP3*	AT3G55370	9.15	36527.36	E(2.96)
*CmDOF14*	KT235688	320	*AtDOF3.6 OBP3*	AT3G55370	9.41	34575.03	N(6.29)
*CmDOF15*	KT235689	338	*AtDOF5.3 TMO6*	AT5G60200	9.1	36783.55	E(2.80)
*CmDOF16*	KT235690	233	*AtDOF5.4 OBP4*	AT5G60850	6.21	25970.76	N(8.62)
*CmDOF18*	KT235692	291	*AtDOF5.4 OBP4*	AT5G60850	8.5	32099.06	N(7.02)
*CmDOF19*	KT235693	214	*AtDOF1.7*	AT1G51700	8.82	23485.19	N(6.32)
*CmDOF20*	KT235694	189	*AtDOF1.7*	AT1G51700	7.11	20784.27	N(7.82)
*CmDOF21*	KT235695	407	*AtDOF5.2 CDF2*	AT5G39660	8.37	44743.54	N(8.86)

*PI, isoelectric point; MW, molecular weight; N, nucleus; E, extracellular*.

**Figure 1:**
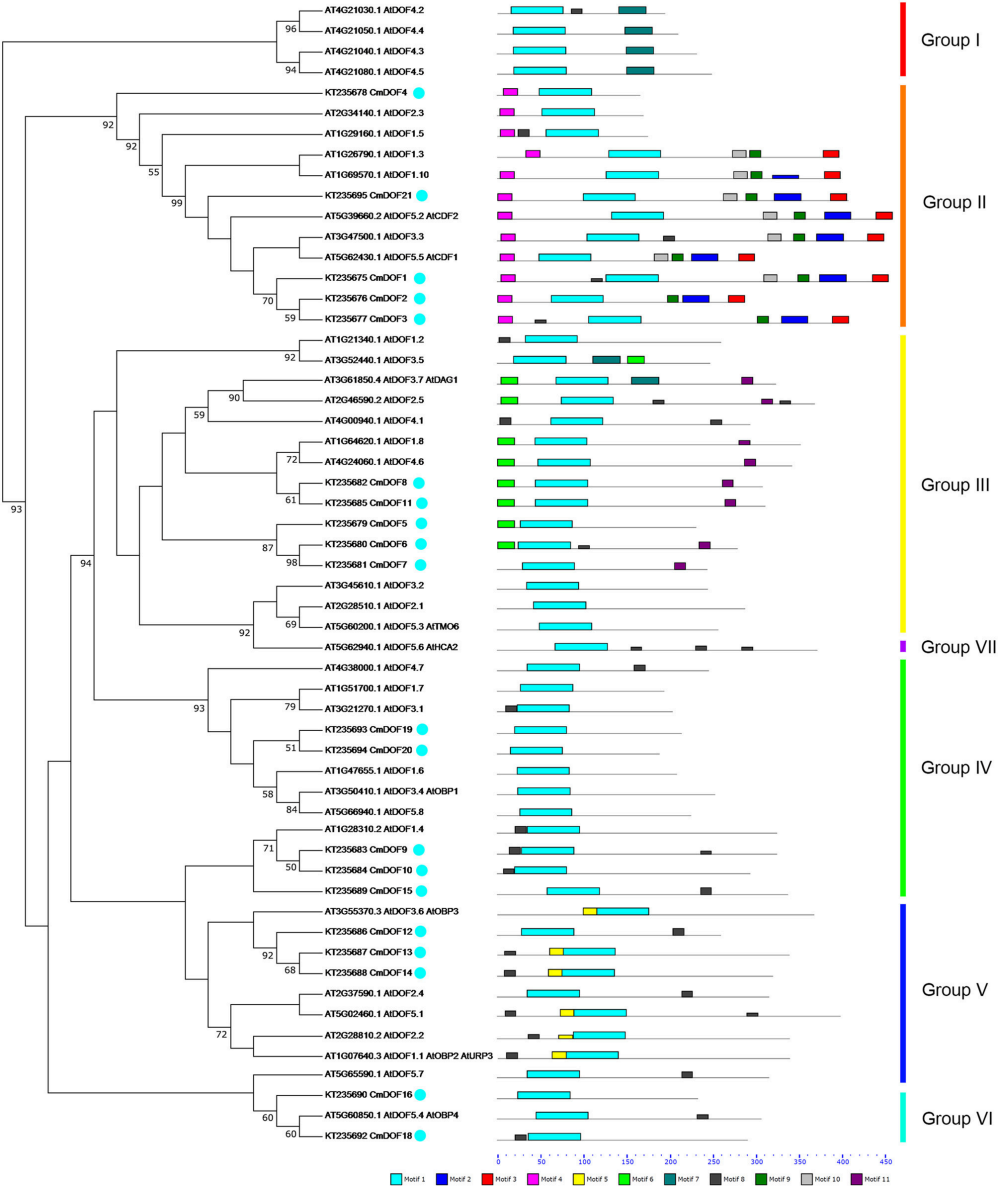
**Phylogenetic tree and distribution of conserved motifs for the *Arabidopsis* and chrysanthemum DOF proteins**. The tree was constructed from a complete alignment of 36 *Arabidopsis* and 20 chrysanthemum DOF proteins using the neighbor-joining method, bootstrap values of greater than 50% are shown at the nodal branches. The right portion shows the distribution of conserved motifs in *Arabidopsis* and chrysanthemum DOF proteins.

To evaluate the evolutionary relationship between *Arabidopsis* and chrysanthemum DOF proteins, the deduced amino acid sequences of the DOF genes identified in *Arabidopsis* and chrysanthemum were completely aligned. A combined phylogenetic tree (Figure 1) was then constructed using the neighbor-joining method and bootstrap analysis (1000 reiterations). Twenty *CmDOF* genes were distributed across five of seven DOF groups: Group II, III, IV, V, and VI. Furthermore, one pair of putative orthologous proteins was identified in *Arabidopsis* and chrysanthemum: AtDOF5.4 with CmDOF18. In contrast, six pairs of paralogous DOF family proteins were identified in chrysanthemum: CmDOF2 and CmDOF3, CmDOF6 and CmDOF7, CmDOF8 and CmDOF11, CmDOF9 and CmDOF10, CmDOF13 and CmDOF14, and CmDOF19 and CmDOF20.

### Conserved sequences in DOF proteins

**Figure 2:**
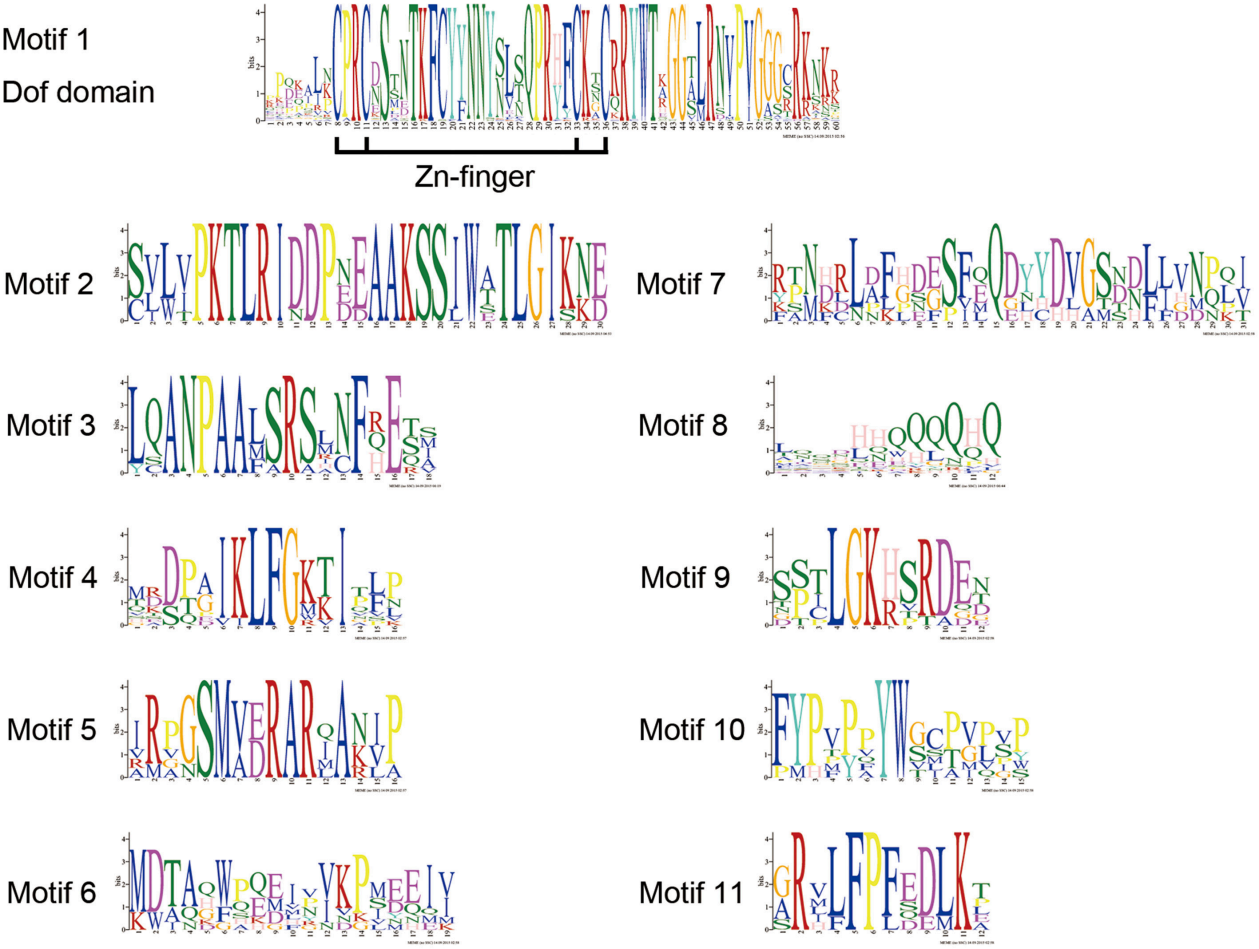
**DOF protein motifs as derived by MEME analysis**. The sequences of motifs of DOF proteins shown in Figure 1.

DOF TFs contain the DOF DNA-binding domain that is usually located near the N-terminal region of the protein. DOF domains were present in all of the deduced DOF proteins in *Arabidopsis* and chrysanthemum based on MEME analysis, and each domain sequence contained ~56 amino acid residues (Motif 1, as shown in Figures 1, 2).

We also identified 10 other motifs shared by *Arabidopsis* and chrysanthemum; for example, Motifs 2/3/4/9 were only shared in Group II, Motif 5 was only present in Group V, and Group III had one specific motif, Motif 6 (Figure 1). Details on these motif features are shown in Figure 2. The Zn finger-like structure is the string CX_2_CX_21_CX_2_C type, which binds zinc (Zn^2+^) (Figure 2).

### miRNA target site prediction and validation

**Figure 3:**
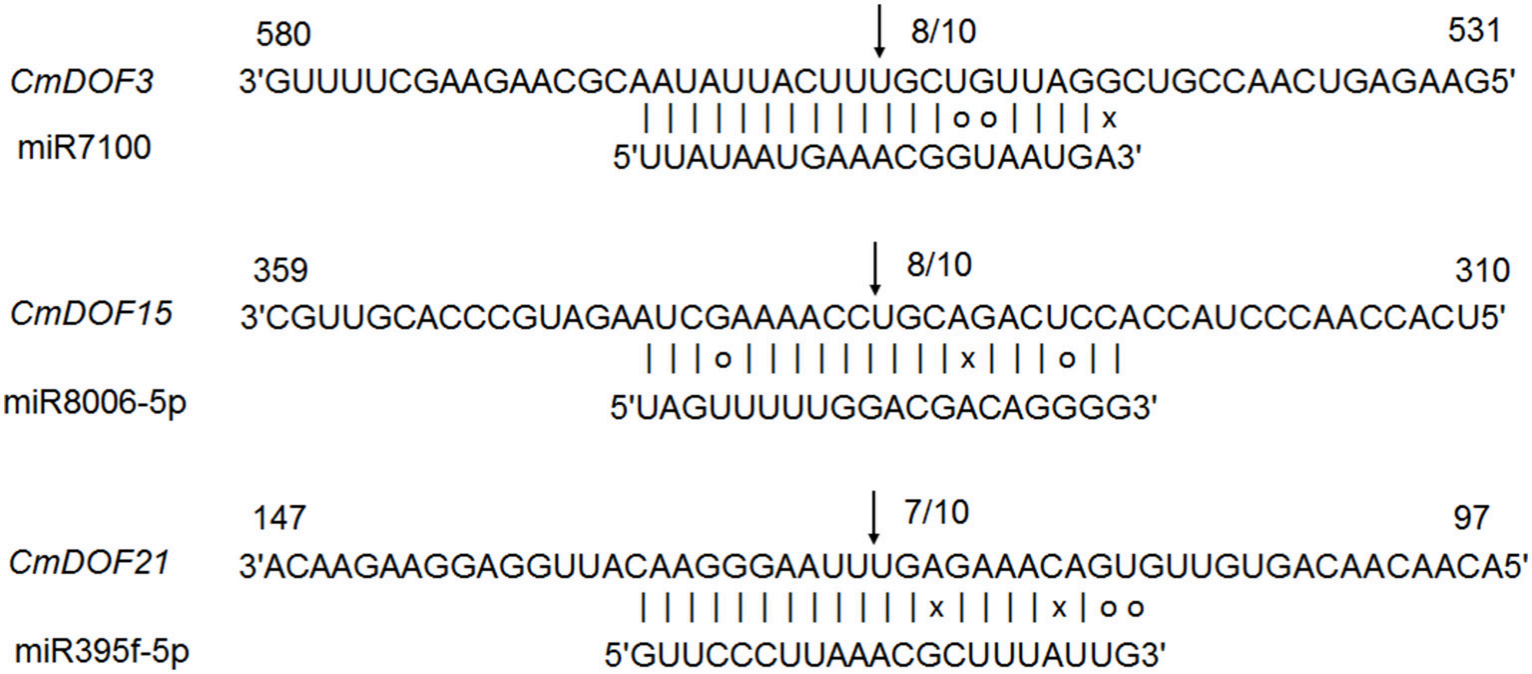
**Mapping of mRNA cleavage sites confirmed by 5′ RLM-RACE**. Arrows indicate the 5′ ends of mRNA fragments, as identified by cloned 5' RLM-RACE products, with the frequency of clones shown.

All plant miRNA data were used to predict target transcript candidates of *CmDOF*s. As shown in Table S4, 13 *CmDOF*s can be targeted by 16 miRNA families. *CmDOF1* has three target sites and *CmDOF21* has two target sites, whereas the other 11 *CmDOF*s (*3, 5, 6, 8, 9, 11, 15, 16, 18, 19*, and *20*) have only one target site. Moreover, we used 5′ RLM-RACE to map the cleavage sites in three predicted target genes. *CmDOF3, 15* and *21* were confirmed as real targets of miRNA, as all of the 5′ ends of the mRNA fragments mapped to the nucleotide that paired to the tenth nucleotide of each miRNA with higher frequencies than depicted for each pairing oligo (Figure 3).

### Transcription profiling of *CmDOF* genes

**Figure 4:**
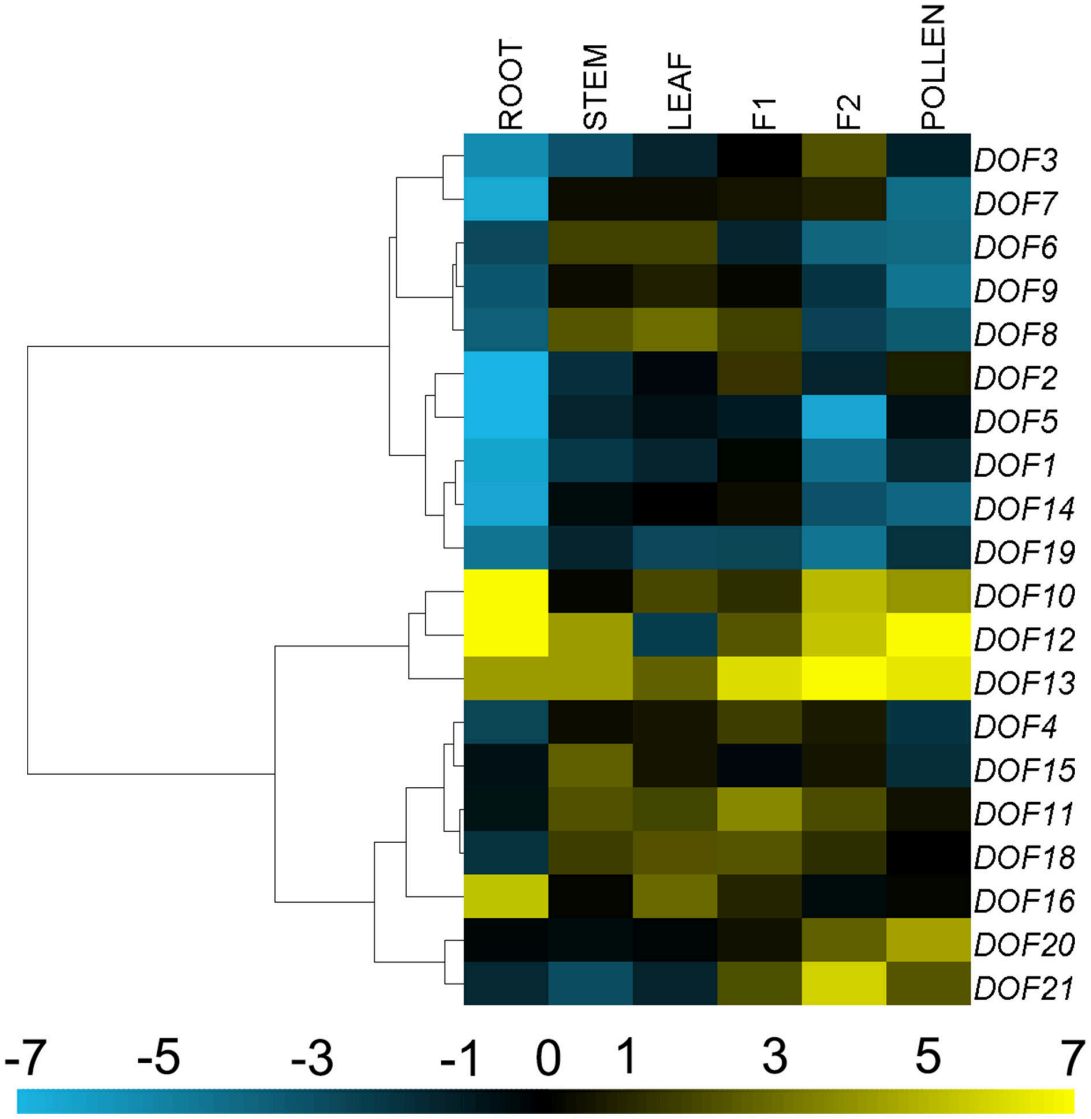
**Differential transcription of *CmDOF* genes**. F1, tubular florets; F2, ray florets at budding stage. Blue and yellow indicate lower and higher transcript abundance, respectively, compared to the relevant controls. Grey blocks indicate that transcription was not detected.

The 20 *CmDOF* genes were differentially expressed throughout the plant (Figure 4). The expression of *CmDOF13* in ray florets was more than four orders of magnitude higher than that of *CmDOF5* in the roots, whereas the *CmDOF9* transcript was not detectable in the ray florets. The expression of *CmDOF20* and *CmDOF21* was significantly higher in reproductive organs than that in vegetative organs, whereas *CmDOF16* was only highly expressed in roots. Interestingly, the expression patterns of six pairs of paralogous *CmDOF* genes were completely different from one another, with the exception *CmDOF6* and *CmDOF7*, which exhibited similar expression patterns.

### Expression of *CmDOF* genes in plants challenged with phytohormones

**Figure 5:**
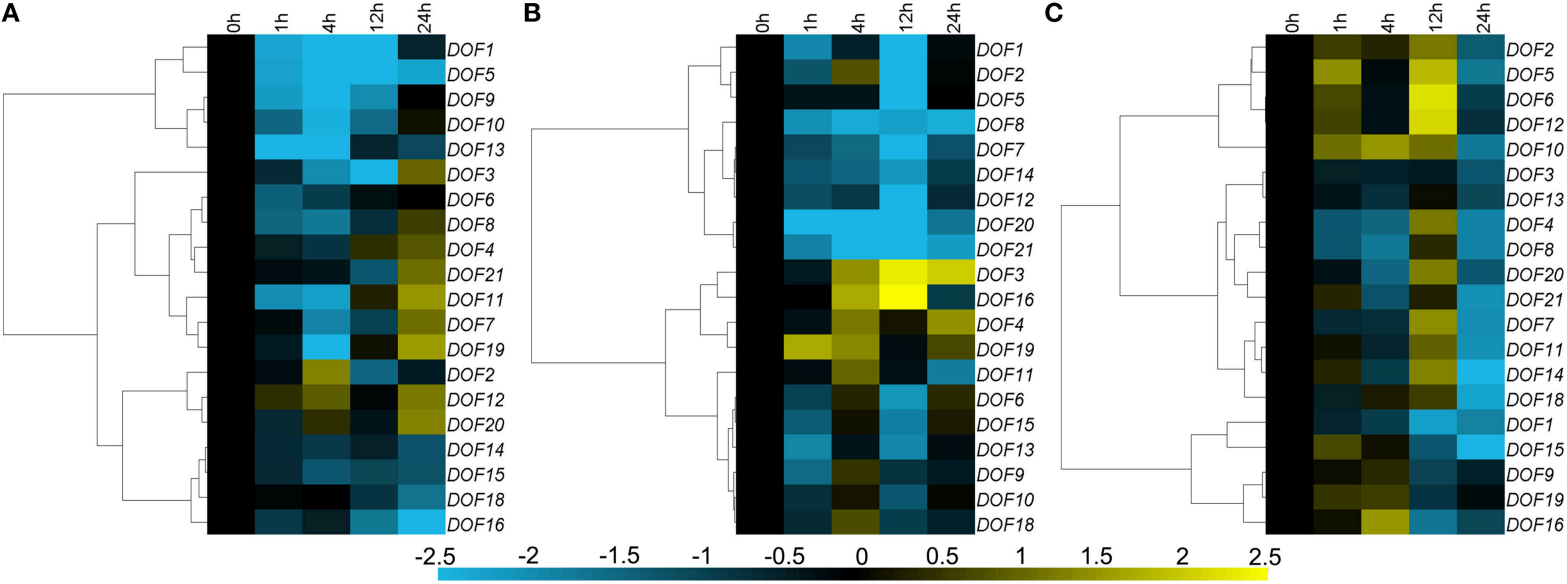
**Differential transcription of *CmDOF* genes in leaves as induced by the exogenous supply of (A) abscisic acid (ABA), (B) methyl jasmonate (MeJA), and (C) salicylic acid (SA) treatments**. Blue and yellow indicate lower and higher transcript abundance, respectively, compared to the relevant controls.

Seventeen of twenty *CmDOF* genes were significantly down-regulated by exogenous ABA, although *CmDOF3, 4, 7, 8, 11, 19*, and *21* were induced at 12 h. The *CmDOF12* and *CmDOF20* transcripts were increased at 4 and 24 h after ABA treatment, whereas *CmDOF2* was only induced at 4 h (Figure 5A). The chrysanthemum *DOF* family genes exhibited three main expression patterns under MeJA treatment. *CmDOF3, 4, 16*, and *19* were induced by the treatment, whereas *CmDOF1, 2, 5, 7, 8, 12, 14, 20*, and *21* were repressed. The transcripts of the other seven *CmDOFs* were decreased at 1 h, increased at 4 h, and decreased at 12 h (Figure 5B). Eighteen of the genes were significantly repressed after 24 h of exposure to SA, whereas the expression of *CmDOF9* and *19* was not significantly influenced by SA. Five genes (*CmDOF2, 5, 6, 10*, and *12*) were induced at 1 and 12 h. The degree of inhibition of *CmDOF1* increased with a longer processing time (Figure 5C).

### Differential responses of the *CmDOF* genes to abiotic stress

**Figure 6:**
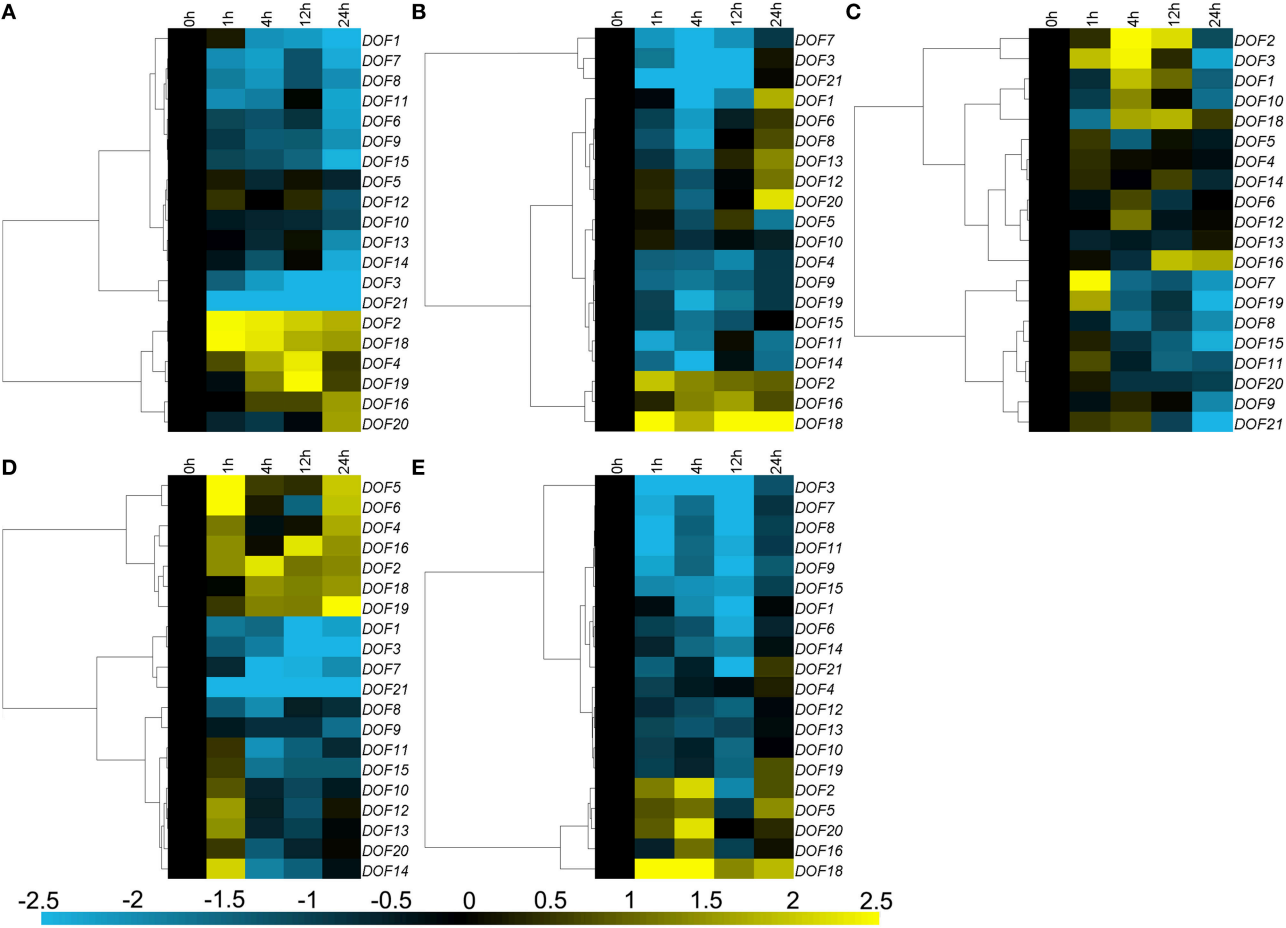
**Differential transcription of *CmDOF* genes in leaves as induced by (A) salinity stress, (B) moisture stress, (C) low temperature (4°C), (D) high temperature (40°C), and (E) wound treatments**. Blue and yellow indicate lower and higher transcript abundance, respectively, compared to the relevant controls.

Three main expression patterns of *CmDOF*s were observed under salinity stress treatment. Eleven *CmDOFs* (*1, 3, 6, 7, 8, 9, 11, 13, 14, 15*, and *21*) were suppressed, whereas five *CmDOF*s (*5, 10, 12, 16*, and *20*) were weakly regulated by salinity stress, with a range of variation of less than 2-fold. Furthermore, *CmDOF2, 4, 18*, and *19* were up-regulated (Figure 6A). The expression of *CmDOF5, 6, 8, 10, 12, 13*, and *20* was not significantly altered by PEG treatment. Moisture stress up-regulated *CmDOF2, 16*, and *18* and markedly suppressed the transcription of 10 other *CmDOF*s (Figure 6B). Twelve of the 20 *CmDOF* genes (*1, 2, 3, 7, 8, 9, 10, 11, 15, 19, 20*, and *21*) were suppressed by exposure to low temperature at 24 h. The transcript abundance of *CmDOF7* and *19* was increased at 1 h and that of *CmDOF1, 2, 3*, and *10* was increased at 4 h. CmDOF16 was induced at 12 and 24 h, whereas the other seven *CmDOF*s (*4, 5, 6, 12, 12, 14*, and *18*) were not significantly affected by low temperature (Figure 6C). Five *CmDOF* genes (*1, 3, 7, 8*, and *21*) were down-regulated by high temperature, whereas seven *CmDOF* genes (*2, 4, 5, 6, 16, 18*, and *19*) were up-regulated. *CmDOF12, 13*, and *14* were induced by high temperature treatment at 1 h but were then repressed thereafter. The other five *CmDOF*s (*9, 10, 11, 15*, and *20*) were not significantly affected by low temperature (Figure 6D). All of the genes, with the exception of *CmDOF2, 5, 16, 18*, and *20*, were down-regulated by mechanical damage (Figure 6E).

## Discussion

The *DOF* genes, members of the plant-specific TF family, are ubiquitous in photosynthetic organisms, ranging from green unicellular algae to vascular plants, and are implicated in important biological processes in plants. The function and evolution of *DOF* genes have been identified in *Arabidopsis* (Gupta et al., 2015), rice (Gaur et al., 2011), maize (Chen and Cao, 2014), poplar (Yang and Tuskan, 2006), *Brachypodium distachyon* (Hernando-Amado et al., 2012), bread wheat (Dong et al., 2007), and sorghum (Kushwaha et al., 2011). Nevertheless, little is known about the chrysanthemum DOF family. In this study, comparative analysis of the DOF family between *Arabidopsis* and chrysanthemum allowed for the prediction of various functions of the chrysanthemum DOF family members and helped to facilitate further gene function analysis.

### Comparative analysis of the chrysanthemum and *Arabidopsis DOF* gene families

In this study, 20 *CmDOF* genes were identified in chrysanthemum based on transcriptome data and were classified by the presence of a highly conserved DOF domain. Gene duplication and differentiation have long been viewed as the major pathways of origin for new genes and for the differentiation of gene function. Therefore, to clarify the phylogenetic relationships among the *CmDOF* genes and infer the evolutionary history of this gene family, a combined phylogenetic tree was constructed based on the alignment of *Arabidopsis* and chrysanthemum DOF sequences (Figure 1). Phylogenetic analysis indicated that there are five subgroups in the transcriptome data. The results suggested that chrysanthemum has other unknown *DOF* genes, which may not have been identified here due to the limited available transcriptome data for chrysanthemum. Here, we also detected six pairs of paralogous *CmDOF* genes and one pair of putative orthologues based on phylogenetic analysis (Figure 1). Orthologues are defined as genes in different genomes that have been created by the splitting of taxonomic lineages, and paralogs are genes in the same genome created by gene duplication events (Thornton and DeSalle, 2000). Paralogs usually display different functions, whereas orthologues may retain the same function (Tatusov et al., 1997).

### Motifs analyses of the *DOF* family in chrysanthemum

We further analyzed the conserved motifs in the chrysanthemum DOF family using the MEME program. The majority of the CmDOFs in the same group shared similar motifs, suggesting that these conserved motifs play crucial roles in group-specific functions. However, high divergence in their structures was found between the different groups. For example, Group II contains Motifs 2, 3, 4, and 9, whereas Group III contains Motifs 6 and 11 (Figure 1), reflecting the complex nature of the function of DOF proteins in chrysanthemum. We also found motifs conserved in certain groups, e.g., Motif 7 in Group I, Motif 11 in Group III, and Motif 5 in Group V. The motif distribution indicated that the genes containing the same motifs were likely produced via gene expansion within the same groups. In total, 41 conserved motifs were identified in poplar, *Arabidopsis*, and rice DOF protein sequences (Yang and Tuskan, 2006). After comparison, we found that most motifs (except Motif 8) are shared by chrysanthemum, poplar, *Arabidopsis*, and rice.

The DNA-binding domain in CmDOF includes a C2C2-type zinc-finger like motif, although the amino acid sequence of this domain is largely different from those of other zinc-finger domains (Figure 2). The cysteine residues for putative coordination of zinc are shown in the DOF domain amino acid sequence. TFs sometimes contain multiple DNA-binding domains. For example, plant-specific WRKY TFs possess different numbers of WRKY DNA-binding domains, which allows the proteins to be classified into subgroups (Song et al., 2014b). However, in the case of DOF proteins, only a single copy of the DOF domain can consistently be found in their N-terminal regions (Figure 1).

### miRNA target site prediction and validation

To our knowledge, reports on miRNA-DOF interactions have been rare. We predicted that 13 *CmDOF*s could be targeted by 16 miRNA families, respectively (Table S4). We also confirmed that *CmDOF3, 15*, and *21* were real targets of the miRNA in plants (Figure 3). However, the function of these interaction regulatory networks in plants should be determined by further research.

### Organ-preferential expression of *CmDOF* genes

Because gene expression patterns can provide important clues for gene function, we used qRT-PCR to examine the expression of *CmDOF* genes in young seedling roots, stems and leaves as well as in the tube and ray florets of inflorescences at the bud stage and in pollen (Figure 4). The expression profiles reveal spatial variations in the expression of *CmDOF*s in different organs. Five pairs of paralogous genes, except for *CmDOF6* plus *CmDOF7*, showed distinct expression patterns, suggesting that significant functional divergence might occur after duplication events.

*CmDOF10, 12, 13*, and *16* showed relatively high expression levels in roots. Among them, only *CmDOF16* was highly expressed in the root, indicating that it could play a role in the development of the plant root. These expression patterns were similar to those of their homolog in *Arabidopsis, AtOBP3*, which regulates phytochrome and cryptochrome signaling (Ward et al., 2005). Expression of *CmDOF20* and *CmDOF21* was significantly higher in reproductive organs than that in vegetative organs, indicating that they could play key roles in reproductive development. The *CmDOF21* homolog *AtCDF2* regulates the timing of transition from the vegetative to reproductive phase (Fornara et al., 2009). The expression level of *CmDOF2* was higher in tube florets, whereas that of the paralogous gene *CmDOF3* was higher in ray florets, suggesting that these genes could play different roles in chrysanthemum flower development. The highly expressed or differentially expressed *CmDOF* genes reported in this study may play a regulatory role in chrysanthemum plant development. However, additional research is needed to determine the functions of the *CmDOF* genes.

### The expression profiles of *CmDOF* genes under phytohormone and abiotic stress treatments

Some plant hormonal signals, such as ABA, SA, and MeJA, are involved in the response to various stresses through activation of the transcription of several defense-related genes. For example, SA and MeJA co-ordinately play a critical role in biotic stress signaling upon pathogen infection (Vos et al., 2015), while ABA is extensively involved in the response to various biotic and abiotic stresses, including pathogen infection, cold, and osmotic stress (Lim et al., 2015). Therefore, in this study, we investigated the responses of *CmDOF*s to different plant hormone signals and abiotic stress treatments. The results showed that *CmDOF*s were both up-regulated and down-regulated by the treatments (Figures 4, 5), indicating that *CmDOF*s might be involved in the responses to various plant hormones that signal a stress response.

In previous reports, DOF proteins have been shown to be regulators of plant hormone-responsive genes and have been shown to mediate the response of gibberellins and auxins (Gupta et al., 2015). The rice DOF protein OsDof3 might be a mediator of GA signaling during germination (Washio, 2001). NtBBF1, which is a DOF protein known to play a pivotal role in regulating *rolB* expression, might provide the possible mechanism of auxin induction (Baumann et al., 1999). *OBP3* (*AtDof3.6*) is induced by SA (Kang and Singh, 2000), although its homologs in chrysanthemum exhibited a different expression pattern. *CmDOF12* was induced by SA, whereas *CmDOF13* and *CmDOF14* were not (Figure 5C). However, little is known about the role of the DOF gene family in ABA and JA hormonal signaling pathways. Our results may provide the basis for advancing research on DOF family genes in stress phytohormone signaling.

A group of five tomato *DOF* genes that are homologous to *Arabidopsis* Cycling DOF Factors (CDFs) function as transcriptional regulators involved in responses to drought and salt stress and flowering-time control in a gene-specific manner (Corrales et al., 2014). *SlCDF1–5* genes were differentially induced in response to osmotic, salt, heat, and low-temperature stresses (Corrales et al., 2014). In chrysanthemum, *CmDOF1* is homologous to *AtCDF2, CmDOF2*, and *CmDOF21* are homologous to *AtCDF1*, and *CmDOF3* is homologous to *AtCDF3* (Table 1). However, they have different expression patterns in the presence of abiotic stress. *CmDOF2* was induced by stress, whereas *CmDOF1, 3*, and *21* were repressed. This result indicated that chrysanthemum homologs of *Arabidopsis* CDF might have various roles in abiotic stress. As very few studies have investigated the role of DOF genes in the plant stress response, this work will lay the foundation for further investigations regarding the role of DOF in the stress response.

## Conclusions

This study is the first transcriptome-wide analysis of the DOF TF family in chrysanthemum. The expression of 20 *CmDOFs* in response to a range of phytohormones and abiotic stress treatments was characterized. In addition, *CmDOF3, 15*, and *21* were confirmed as real targets of miRNA in plants. These findings lay the foundation for future research on the function of *CmDOF* genes in the plant stress response, which will promote their application in chrysanthemum breeding.

## Author contributions

Conceived and designed the experiments: AS, SC, and FC. Performed the experiments: AS, TG, JX, QF, and KZ. Analyzed the data: AS, SC, and FC. Contributed reagents/materials/analysis tools: ZG and FC. Wrote the paper: AS, PL, and DW. All authors read and approved the final manuscript.

### Conflict of interest statement

The authors declare that the research was conducted in the absence of any commercial or financial relationships that could be construed as a potential conflict of interest.
